# The emissions of CO_2_ and other volatiles from the world’s subaerial volcanoes

**DOI:** 10.1038/s41598-019-54682-1

**Published:** 2019-12-10

**Authors:** Tobias P. Fischer, Santiago Arellano, Simon Carn, Alessandro Aiuppa, Bo Galle, Patrick Allard, Taryn Lopez, Hiroshi Shinohara, Peter Kelly, Cynthia Werner, Carlo Cardellini, Giovanni Chiodini

**Affiliations:** 1 Department of Earth and Planetary Sciences, University of New Mexico, Albuquerque, NM 87131 USA; 2 Department of Space, Earth and Environment, Chalmers University of Technology, SE-412 96 Göteborg, Sweden; 3 Department of Geological and Mining Engineering and Sciences, Michigan Technological University, Houghton, MI 49931 USA; 4 Dipartimento di Scienze della Terra e del Mare (DiSTeM), Università di Palermo, 36 90123 Palermo, Italy; 5 Institut de Physique du Globe de Paris (IPGP), Université de Paris, 75005 Paris, France; 6 Geophysical Institute, Alaska Volcano Observatory, University of Alaska Fairbanks, Fairbanks, AK 99775 USA; 7 Geological Survey of Japan, AIST, Tsukuba, 305-8567 Japan; 8 U.S. Geological Survey, David A. Johnston Cascades Volcano Observatory, Vancouver, WA 98683 USA; 9 Dipartimento di Fisica e Geologia, Università di Perugia, Perugia, Italy; 10 INGV, Sezione di Bologna, Bologna, Italy

**Keywords:** Solid Earth sciences, Chemistry

## Abstract

Volcanoes are the main pathway to the surface for volatiles that are stored within the Earth. Carbon dioxide (CO_2_) is of particular interest because of its potential for climate forcing. Understanding the balance of CO_2_ that is transferred from the Earth’s surface to the Earth’s interior, hinges on accurate quantification of the long-term emissions of volcanic CO_2_ to the atmosphere. Here we present an updated evaluation of the world’s volcanic CO_2_ emissions that takes advantage of recent improvements in satellite-based monitoring of sulfur dioxide, the establishment of ground-based networks for semi-continuous CO_2_-SO_2_ gas sensing and a new approach to estimate key volcanic gas parameters based on magma compositions. Our results reveal a global volcanic CO_2_ flux of 51.3 ± 5.7 Tg CO_2_/y (11.7 × 10^11^ mol CO_2_/y) for non-eruptive degassing and 1.8 ± 0.9 Tg/y for eruptive degassing during the period from 2005 to 2015. While lower than recent estimates, this global volcanic flux implies that a significant proportion of the surface-derived CO_2_ subducted into the Earth’s mantle is either stored below the arc crust, is efficiently consumed by microbial activity before entering the deeper parts of the subduction system, or becomes recycled into the deep mantle to potentially form diamonds.

## Introduction

Volcanism is the main pathway for the transfer of carbon and other volatiles stored in the deep Earth to the surface^1,2^. Volcanic carbon dioxide (CO_2_) is a key non-anthropogenic regulator of atmospheric CO_2_ levels and has, over geologic time scales, affected the evolution of Earth’s climate^3–5^. Quantifying the volcanic CO_2_ flux is an ongoing challenge for the volcano science community and was the primary driver for launching the Deep Earth Carbon Degassing (DCO-DECADE) initiative of the Deep Carbon Observatory. DCO-DECADE was first established in 2011 during IAVCEI Commission of the Chemistry of Volcanic Gases workshop in Kamchatka and has since provided unprecedented global coverage of volcanic gas emission and composition measurements through continuous monitoring and campaign efforts (https://en.wikipedia.org/wiki/Deep_Earth_Carbon_Degassing_Project).

Here we present the results of this initiative to date, providing a comprehensive assessment of the present-day (2005–2015) global volcanic CO_2_ flux to the atmosphere. We combine measured volcanic fluxes of sulfur dioxide (SO_2_) with measured or estimated C/S molar ratios to derive volcanic CO_2_ fluxes, as initially done by Allard *et al*.^6^ and Williams, *et al*.^7^. These data are then extrapolated to volcanoes world-wide. Our present update greatly benefits from recent advances in satellite data analysis and a global compilation of annual emissions measured by the Ozone Monitoring Instrument, OMI^8^, which provides SO_2_ flux data for numerous volcanoes simultaneously. This space-based record is complemented by and compared to ground-based SO_2_ flux data, particularly from the Network for Observation of Volcanic and Atmospheric Change NOVAC^9^, to obtain annual budgets of global volcanic SO_2_ emissions. At the same time, coverage of C/S ratios from degassing volcanoes has significantly improved with the ubiquitous deployment of Multicomponent Gas Analysis Systems (MultiGAS) providing such data over wide temporal and spatial scales^10^. We focus primarily on degassing from subaerial volcanic vents but we also include a preliminary evaluation of diffuse soil degassing from the volcanoes’ flanks or from active tectonic regions, which has been compiled in the Magmatic Degassing (MaGa) database (www.magadb.net) and just reported in Werner, *et al*.^11^.

## Data and Method

Emissions from subaerial volcanoes with plumes were compiled and evaluated by the DCO-DECADE synthesis group to produce an updated and complete estimation of global volcanic SO_2_ and CO_2_ fluxes. Our compilation applies to approximately 900 volcanoes, listed by Syracuse and Abers^12^. Most of them are located in subduction zones and are documented for subduction-related geophysical parameters, which thus offers the opportunity to compare our results for volcanic output to subduction forcing functions. We also consider volcanoes not listed in Syracuse and Abers but documented for SO_2_ or CO_2_ fluxes, these being mainly from hot spot and continental rift localities.

**Figure 1:**
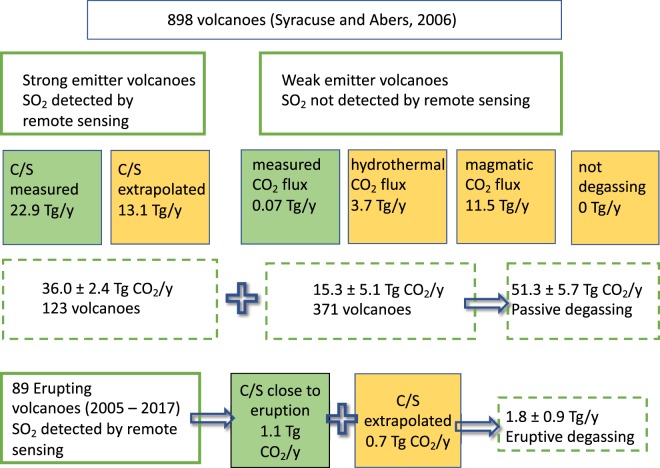
Schematic diagram of how fluxes were calculated and estimated. Green fields indicate measured values, yellow fields indicate estimated or extrapolated values. Numbers are the results as discussed in text with final uncertainties.

The presented dataset includes volcanic emissions from both persistent strong emitters and discrete eruptions, but also provides new estimates for the emissions of  weak contributors. Strong emitters are defined as volcanoes that release greater than 0.014 TgSO_2_/y as detected by satellite. Emissions from erupting volcanoes are identified by using the Global Volcanism Program catalogue for eruptive activity between 2005 and 2017. Weak contributors have SO_2_ emissions below OMI’s detection limit (0.014 TgSO_2_/y^8^). The extrapolations to unmeasured volcanoes utilize new data on C/S ratios and a classification into magmatic and hydrothermal categories based on visual observations, volcano databases (predominantly from Smithsonian Institution’s Global Volcanism Program, 2013), field reports, and observations made by the authors. Note that while we report and discuss molar C/S ratios, we use mass ratios to compute CO_2_ mass fluxes from SO_2_ mass fluxes. The summary methodology of computing and extrapolating the fluxes is shown in Fig. 1.

### SO_2_ flux from strong emitters and during eruptions

Our compilation of SO_2_ flux data covers 11 years from 2005 to 2015 and uses information from long-term monitoring from space and ground, as well as short-term campaign data and reports from the literature. When contemporaneous satellite- and ground-based (long-term or campaign) data exist for the same volcano, we made a critical case-by-case selection of the best and most representative estimate of the annual emission, considering the coverage of each method, specific conditions of measurement and, in certain cases, the separation of the contributions from two neighboring volcanoes that cannot be separated by satellite observations alone. In 28 out 125 cases, ground-based measurements are the only measurements available and are used for the calculations. Therefore, in order to be internally consistent, we used satellite-based measurements where possible and ground-based measurements only when satellite observations were not available. From the OMI survey the global SO_2_ flux from persistent volcanic degassing in the period 2005–2015 is estimated as 23.2 ± 2 Tg SO_2_/y (±1 s.d.) for ~90 measured volcanoes (Table S1 and ref. ^8^). This includes SO_2_ from large emitters such as Anatahan (1335 t SO_2_/d or 0.5 Tg/y), Bagana (3775 t SO_2_/d or 1.4 Tg/y), Aoba (2870 t SO_2_/d or 1 Tg/y) and Manam (1484 t SO_2_/d or 0.5 Tg/y). Here we complement this satellite-based estimate of decadal emission data with data from the ground-based NOVAC SO_2_ flux network^9,13^ and from campaign-style measurements for the same 2005–2015 period. The SO_2_ flux obtained from ground-based measurements is 6.9 ± 0.8 Tg SO_2_/y for 66 volcanoes, some of which were simultaneously observed by OMI. Using the combination approach described above, we account for 125 individual volcanoes in persistent degassing which, altogether, produced 24.9 ± 2.3 Tg SO_2_/y in 2005–2015 (Table S1).

The SO_2_ flux released during explosive and effusive eruptions is also compiled in Table S1. The time period considered for eruptions extends from 2005 to 2017 and is based on the SO_2_ climatology produced by NASA (https://so2.gsfc.nasa.gov/index.html). During this 13-year time period eruptions from 89 volcanoes were observed and emitted a total of 33.3 Tg SO_2_. This corresponds to an average global eruptive flux of 2.6 Tg SO_2_/y, ∼10 times lower than the annual flux from global persistent degassing. A similar proportion was previously assessed^14,15^, despite differences in absolute figures. Therefore, our best estimate for strong volcanic emitters that have been measured either by satellite or by ground-based techniques during both persistent and eruptive degassing is 27 ± 2 Tg SO_2_/y.

### CO_2_ flux from Strong Emitters

Our study builds on previous work by Werner, *et al*.^11^ and incorporates additional volcanoes with SO_2_ flux constraints by OMI and by ground-based techniques. Here we consider the 125 volcanoes which, as described above, had their SO_2_ flux measured from space and/or from the ground. Among these, 67 have measured C/S ratios (Table S1). As shown by^16^, in assessing volcanic CO_2_ fluxes it is critical to use C/S ratios that are representative for the magmatic gas component and not or negligibly affected by sulfur removal during low-temperature hydrothermal processes. In addition to the C/S ratios reported and scrutinized in^10,17^, we here report C/S ratios for 10 more volcanoes. These ratios were measured with MultiGAS in crater plumes, thereby minimizing low temperature hydrothermal influences and meeting the requirements proposed by^10^. While C/S ratios have been shown to vary with volcanic activity, long-term average ratios correlate with petrologic indicators and therefore are thought to represent magmatic source compositions. Following the approach of^10^, we thus averaged the available gas data for each volcano to obtain a mean ± 1 SD (1 standard deviation) CO_2_/SO_2_ ratios and effectively integrate the “time-averaged” degassing of each volcano. This procedure minimizes the weight of transient phases of either CO_2_enriched or, instead, CO_2 _depleted volcanic gases that are typically observed either prior to or after eruption, owing to the contrasted solubilities of CO_2_ and S in magmas (e.g.^18,19^) and the earlier degassing of CO_2_ during magma ascent and decompression. We note, moreover, that such sequential degassing steps average out in the long-term, as magmas are convectively transported from their deep (CO_2_-degassing favored) to near-surface (S-degassing favored) storage zones (eg.^20–22^). Mafic to intermediate volcanoes typically erupt more than once in a decade. Over timescales of years/decades, therefore, we stress that time-averaged volcanic gas compositions will most closely approach the CO_2_/S proportions in the primary undegassed magma and that of the magma source^23,24^. The error analyses of our results are discussed separately below. For the 67 volcanoes with measured SO_2_ fluxes and representative magmatic C/S ratios we obtain a total flux of 22.9 Tg CO_2_/y. An additional 8 volcanoes have measured C/S ratios but no SO_2_ flux constraints. In order to estimate the CO_2_ flux from the remaining volcanoes that have measured SO_2_ fluxes (among a total of 125), we need an indirect approach to assess unmeasured magmatic C/S ratios. Aiuppa, *et al*.^17^ have shown that magma’s geochemical signatures can be used to predict magmatic C/S ratios for arc volcanoes that are located in a same arc segment. The 35 predicted ratios directly taken from Aiuppa, *et al*.^17^ are shown in Table S1. For 33 of these volcanoes with measured SO_2_ flux we infer a cumulative CO_2_ flux of 11.1 Tg CO_2_/y. To estimate C/S ratios of the remaining 24 volcanoes we again adopt the approach of^17^ where unmeasured volcanoes are categorized in Groups 1, 2 and 3 based on their tectonic location. Group 1 and 2 volcanoes have respective average C/S ratios of 1.2 ± 0.5 and 2.4 ± 0.7 (range: 2.1 ± 0.7 to 2.7 ± 0.7) that primarily reflect the C input from subducted slab-derived fluids, whereas Group 3 volcanoes receive substantial additions of carbon from the overlying crust and display higher C/S ratios (5 ± 1.5 on average)^17^. Here we distribute volcanoes undocumented for C/S in Groups 1, 2 and 3 according to their tectonic setting and we assign them the above average ratio for each Group. Using this approach, we are able to assign C/S ratios to 61 volcanoes in Table S1. Among these, 23 have a known SO_2_ flux and hence provide a total flux of 2.0 Tg CO_2_/y. Using this approach, we thus obtain CO_2_ fluxes for a total of 123 volcanoes with either measured or estimated C/S ratios. There are only two arc volcanoes, namely Poas in Costa Rica and Iwo-Jima in Japan, with directly reported CO_2_ flux (0.038 Tg CO_2_/y and 0.1 Tg CO_2_/y, respectively)^11^. Table S1 also includes 4 volcanoes with known SO_2_ flux but whose C/S ratio cannot be inferred from the above method since they occur in hot spot or continental rift settings. The hot spot volcano, Kilauea, is an important emitter: its ground-based measured CO_2_ flux averaged 8,587 ± 7,161 t/day (3.1 ± 2.6 Tg/y) in the period 2005–2017^11^, excluding the 2008–2010 phase of heightened eruptive activity. A lower flux of 3,174 ± 1708 t CO_2_/day (1.2 ± 0.6 Tg CO_2_/y) was estimated from solely OMI SO_2_ fluxes and C/S ratios in 2005–2018. The reported flux error is based on a SD of 0.3 for the C/S ratio. As shown by Werner *et al*.^11^, in general there is a quite good agreement between CO_2_ fluxes indirectly derived from ground-based and satellite measurements at those volcanoes covered by both methodologies, with overall only 20% higher values for the ground-based measurements. In the case of Kilauea, the flux difference merely results from the use of a constant C/S ratio of 0.92 combined with the entire OMI SO_2_ flux record, whereas ground-based sensing actually captured a period of anomalously high CO_2_ degassing (high C/S ratio) at the summit in 2005–2007, prior to the 2008 eruption^25^. In order to be consistent with our approach using OMI and C/S ratios for high emitters, we consider here the OMI-based conservative CO_2_ flux of 1.2 Tg CO_2_/y at Kilauea.

In summary, we assess a best estimate of 13.1 Tg CO_2_/y for the cumulative CO_2_ flux from volcanoes whose SO_2_ plume emissions are strong enough to be quantified either from space or from the ground, but whose C/S ratios were not measured. Therefore, the overall persistent (non-eruptive) CO_2_ flux from the 125 volcanoes discussed above is 36.0 Tg CO_2_/y or 8.2 × 10^11^ mol CO_2_/y.

### CO_2_ flux from explosively erupting volcanoes

In order to estimate the CO_2_ flux from explosively erupting volcanoes, it is necessary to know the C/S ratio during such eruptions. But, due to obvious challenges, C/S determinations during such events are scarce (Table S1). One example is the Cotopaxi 2015 eruption, in Ecuador, during which a C/S range of 0.6 to 2.1 was measured^26^. We note that the inferred ratio of 1.8 we used in Table S1 to compute the eruptive CO_2_ flux from this volcano plots well in this range. Although more determinations of eruptive gas ratios are badly needed, we show below that explosive eruptions contribute modestly to global volcanic CO_2_ emissions and, so, that even a factor 2 uncertainty in C/S ratio during an explosive event has a relatively minor bearing. For a small number of volcanoes the C/S ratio has been measured just before or after an explosive eruption. Assuming that this ratio is representative for the eruption, we can estimate a CO_2_ flux for these volcanoes. 26 out of the 89 volcanoes that erupted between 2005 and 2017 have this datum available (Table S1). For these volcanoes we estimate a total eruptive release of 14.5 Tg CO_2_ during the eruptions from 2005–2017, i.e. a mean annual flux of 1.1 Tg CO_2_. If we simply extrapolate the average flux per erupting volcano to all (arc and non-arc) of the 89 volcanoes that erupted in 2005–2017, we obtain a total eruptive release of 49.5 Tg CO_2_ or a flux of 3.8 Tg CO_2_/y. If we instead use Aiuppa *et al*.’s approach^17^ to predict C/S ratios at the 52 undocumented arc volcanoes that erupted in 2005–2017, based on their grouping category, we can estimate an arc eruptive CO_2_ flux of 0.7 Tg CO_2_/y (9.0 Tg CO_2_ in total). The remaining 11 erupting volcanoes are in hot spots or rifts, where we do not know C/S ratios (Table S1). Therefore, our best estimate for eruptive CO_2_ emissions in the period 2005–2017 is 23.5 Tg CO_2_ or about 1.8 Tg CO_2_/y (4.1 × 10^10^ mol CO_2_/y). This is nearly identical to the estimate of 1.6 Tg CO_2_/y for explosive eruptions and 1.9 Tg CO_2_/y for both explosive and effusive eruptions presented in Werner *et al*.^11^. These figures represent only about 5% of the total CO_2_ flux from persistently degassing volcanoes, therefore reiterating the early discovery that quiescent volcanic degassing contributes the bulk of volcanic emissions globally^7,14,15^.

### SO_2_ and CO_2_ fluxes from weak emitters

Out of all approximately 900 volcanoes listed in Syracuse and Abers^12^, there are 125 strong volcanic emitters documented by either OMI or ground-based SO_2_ flux measurements (Table S1). There are also 19 weaker emitting volcanoes, undetected by OMI, but whose CO_2_ flux was determined from either ground-based sensing of SO_2_ flux and C/S ratio (Table S1) or direct CO_2_ measurement. These 19 volcanoes, not listed in Table S1, emit 1.50 TgCO_2_/y. Apart from these 144 volcanoes, there remain 756 volcanoes in Syracuse and Abers’s^12^ inventory for which we have essentially no data and that require extrapolation in order to constrain the global volcanic volatile fluxes (Fig. 1). All these volcanoes either are not degassing at all or are weak emitters of SO_2_ and, therefore, we expect their overall contribution to the global CO_2_ budget to be small.

**Figure 2:**
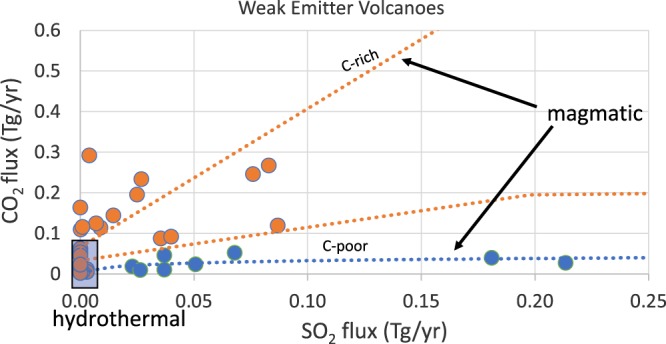
Weak emitter volcanoes SO_2_ detected by ground-based campaigns, CO_2_ either directly measured or determined using ground-based SO_2_ flux measurements and C/S ratios (Table S2).

In addition to the 19 weak emitters mentioned above, we select a number of volcanoes in Table S2 that have low SO_2_ fluxes (<0.1 Tg/y) but well characterized C/S ratios. 38 of these have both measured CO_2_ and SO_2_ fluxes, two volcanoes displaying relatively high SO_2_ flux (Ebeko, 0.18 Tg/y, and Satsuma Iwojima, 0.21 Tg/y). A plot of CO_2_ flux versus SO_2_ flux data for the 38 volcanoes (Fig. 2) reveals two main populations of degassing. The first population, which we define as “hydrothermal”, is characterized by very low SO_2_ flux (<0.003 Tg SO_2_/y or <8 t SO_2_/day) and CO_2_ flux up to ~0.1 Tg/y (~275 t CO_2_/day). SO_2_ fluxes below the limit of 8 t SO_2_/day are exceedingly difficult to measure, even with ground-based techniques, and point to a very low magmatic gas supply, which justifies that the corresponding volcanoes be categorized as non-magmatic or hydrothermal degassers. The 275 t CO_2_/day value (the highest observed) reflects the fact that low-temperature hydrothermal gas emissions can have exceedingly high C/S ratios^10^. The second population, defined as “magmatic”, has an SO_2_ flux >0.003 Tg /y and is composed of two distinct sub-groups (C-rich and C-poor) in terms of CO_2_ flux. The low-C group (blue line) has CO_2_ fluxes <0.05 TgCO_2_/y irrespective of the SO_2_ flux. The high-C group has up to about 3 times as much CO_2_ than SO_2_. Two fitting lines are shown for the high-C group, one is a linear fit, the other is an exponential fit. The low-C group is fitted by an exponential fitting curve.

While the above evaluation gives us the general understanding that volcanic CO_2_ emissions fall into two groups (hydrothermal and magmatic), we can use the following statistical approach to compute the probability that a non-measured volcano has a CO_2_ flux value below a determined value that is intrinsic to the population of the weak emitter volcanoes. In order to do this, we use the measured CO_2_ flux of all 45 volcanoes in Table S2 to extrapolate to the remaining volcanoes that have no measurements, in order to obtain the most probable total global volcanic CO_2_ flux.

**Figure 3:**
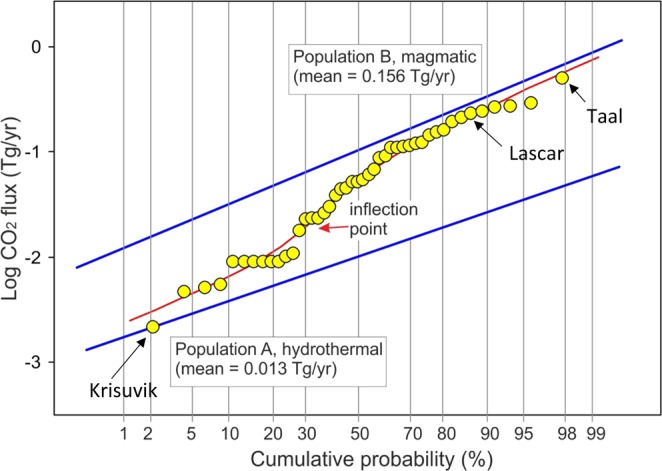
CO_2_ GSA method commonly applied to partitioning complex distribution of soil CO_2_ flux data in different log normal populations^27^ applied to weak emitter volcanoes using data from Table S2. Also shown are several volcanoes that span a range of CO_2_ fluxes. Two populations are identified: population (**A**) hydrothermal with mean CO_2_ flux of 0.013 Tg CO_2_/y and (**B**) magmatic with mean CO_2_flux of 0.156 Tg CO_2_/y. These populations are used to extrapolate to other weak emitter volcanoes for which no data is available.

For our extrapolation to non-measured  weak emitters, we utilize the GSA method commonly applied to partitioning complex distribution of soil CO_2_ flux data in different log normal populations^27^. In this treatment, the first step is to plot the log CO_2_ flux values in a logarithmic probability plot (Fig. 3) where a log normal population plots along a straight line while the combined distribution resulting from the overlapping of n log normal populations plot along a curve characterised by n-1 inflection points. The 45 measured log CO_2_ flux values (yellow circles in Fig. 3) plot along a curve (red line in Fig. 3) with an inflection point at a cumulative probability of 35% indicating the overlapping of the two log normal populations A (fraction 0.35, mean = −2, σ = 0.33) and B (fraction 0.65, mean = −0.99, σ = 0.4). In terms of n log data, the low flux population A (hydrothermal) has a mean of 0.013 Tg/yr (with confidence intervals of 5% and 95% of 0.009 and 0.019 Tg/yr, respectively) while the high flux population B (magmatic) has a mean of 0.156 Tg/yr (with confidence intervals of 5% and 95% of 0.11 and 0.216 Tg/yr, respectively). These results show that hydrothermal fluxes are likely 0.013 TgCO_2_/y, rather than the 0.1 TgCO_2_/y estimated visually in Fig. 2, while the magmatic CO_2_ flux is likely 0.16 Tg CO_2_/y (431 t CO_2_/day) which cannot be estimated from the data plotted in Fig. 2. We emphasize that with more data for weak emitters, this characterization will improve in certainty.

The next step is to determine which of the 756 volcanoes for which there are no data available are either not emitting gas, or belong to the hydrothermal or magmatic CO_2_ flux populations. In order to assess this, we utilized the Global Volcanism Program (GVP) data base, examined recent photographs of these volcanoes to better characterize activity and relied on our combined experiences. The approach that we take is to generally evaluate visually whether an unmeasured volcano is likely to exhibit ‘magmatic’ or ‘hydrothermal’ characteristics. A magmatic gas signature would be recognized by a visible fumarolic plume and/or recent (2005–2017) eruptive activity. A hydrothermal gas signature is assigned for volcanoes that have warm, potentially steaming ground, degassing through mud-pools or water, no coherent plume and no large fumaroles. A volcano that is not active, i.e. not degassing at all, lacks all of the above characteristics.

While arguably subjective, this method provides an estimation of the number of degassing volcanoes world-wide that do not have a detectable SO_2_ emission and allows for evaluating the type of degassing. We classified 404 volcanoes as not degassing, 278 volcanoes that are degassing hydrothermal gas and 74 volcanoes that are degassing magmatic gas (756 total volcanoes), thus giving a total of 371 degassing volcanoes not detected by OMI or in the NOVAC network (Fig. 1).

Ascribing the mean estimated hydrothermal and magmatic flux values to these sets of volcanoes results in 3.7 Tg CO_2_/y degassing from hydrothermal volcanoes and 11.5 Tg CO_2_/y from magmatic volcanoes for a total of 15.2 Tg CO_2_/y for these unmeasured volcanoes globally. Including the mean of 0.07 Tg CO_2_/y of the 19 measured  weak emitters (Table S2) results in a total of 15.2 TgCO_2_/y or 3.5 × 10^11^ mol CO_2_/y for all weak emitters (Table 1, Fig. 1).

**Table 1:** CO_2_ fluxes from weak emitters (SO_2_ not detected by OMI) globally.

	Number of volcanoes	Global CO_2_ flux Tg/yr		
			5%	95%
hydrothermal (A)	278	3.7	2.5	5.2
magmatic (B)	74	11.5	8.1	16.0
non degassing	404	0	0	0
**TOTAL**		**15.2**	**10.6**	**21.2**

### Error assessment

An important consideration when evaluating the fluxes of volcanoes globally is the assessment of the error. Carn *et al*.^8^ provide estimates of the uncertainty in annual mean SO_2_ fluxes measured by OMI (~55%), which includes errors associated with retrieved SO_2_ columns and with variable plume altitude, wind speed, and SO_2_ lifetime. The uncertainty for the ground-based SO_2_ fluxes measured by the NOVAC network and selected for this compilation is below 30%. Table S1 provides the fractional uncertainty of the SO_2_ flux for each volcano. This uncertainty is estimated at 55% and 30% for space- and ground-based measurements, respectively^8,9^. This reported uncertainty of ∼30% for ground-based SO_2_ flux measurements with scanning-DOAS follows from the analyses presented in^9,13,28^. The annual means in SO_2_ fluxes and their standard errors reported here were calculated from the daily means of SO_2_ flux measurements, which in turn were calculated from the individual measurements of flux, when at least five measurements of ‘good quality’ were available in a given day. An individual SO_2_ flux measurement was considered ‘good quality’ when several criteria were met, namely: low spectral fit error in the derivation of SO_2_ column densities, distance to the plume less than 5 km, complete coverage of the scanned plume, use of values calculated by triangulation for plume height, and use of the (then) best available information for plume speed (ECMWF ERA-interim). With these criteria, we consider that our measurements were taken under ‘good conditions’, as defined in^9^. This means relative uncertainties of 10% for spectroscopy, 10% for measurement geometry, 20% for wind speed, and 20% for atmospheric scattering, all added as independent variables in quadrature. The value of 20% for scattering (or radiative transfer effects), is slightly higher than ‘good’, to account for possible effects not accounted for in this analysis. Table S1 provides the fractional uncertainty of the SO_2_ flux for each volcano. The error of CO_2_ flux is strongly dependant on the methods that are used. In our compilation total error estimates for volcanoes with measurements are composed of the errors in the SO_2_ flux measured by OMI or by ground-based techniques and the variability of this flux over the 13-year time period, as well as the errors in the C/S ratios used and their variability over the 13-year time period. SO_2_ flux variability, C/S variability and CO_2_ flux variabilities over the 13-year time period are expressed as a standard deviation in Tables S1 and S2 (one sigma). The errors of our estimates become significantly larger for non-measured volcanoes where extrapolated values are used for C/S ratios based on tectonic setting or when we need to visually categorize volcanoes as degassing or not degassing, and hydrothermal vs. magmatic (Table 2, Fig. 1). For the predicted C/S ratios based on the volcano group and petrologic method, we ascribe a SD of 1.5 for group 3 and 0.8 for Groups 1 and 2, consistent with the SD ascribed in^17^. The summary of all volcanic CO_2_ fluxes is shown in Table 2 and we report the cumulative SD obtained from the SD of the flux SO_2_ flux measurements and the C/S ratios. In order to assess the error of the measured and predicted CO_2_ flux from strong emitters, we apply the Monte Carlo method to the summary of the strong emitter volcanoes CO_2_ data set. In the simulation, the CO_2_ flux for each volcano is set to vary randomly within its mean ± SD value and the resulting CO_2_ fluxes are summed together. This procedure is repeated 100 times, resulting in 100 randomly-generated sums. The total values reported in Table 2 are the ranges (mean ± 1 SD) of 70% of the random generated sums. Using the Monte Carlo approach, the total CO_2_ flux from passively degassing volcanoes then becomes 51.3 ± 5.7 Tg CO_2_/y.

**Table 2:** Results of CO_2_ flux estimates from subaerial volcanoes in the period from 2005 to 2017.

From 2005 to 2017	number	CO_2_ flux Tg CO_2_/y
Strong emitter volcanoes (SO_2_ flux measured by OMI or ground-based)		
with C/S measured	67	22.9
with C/S extrapolated based on petrology (Aiuppa *et al*., in rev)	33	11.1
with C/S extrapolated no petrology available	23	2.0
**Total flux from strong emitter volcanoes**	**123**	**36. 0± 2.4**
Weak emitter volcanoes		
measured CO_2_	19	0.07
ascribed hydrothermal	278	3.7
ascribed magmatic	74	11.5
non degassing	404	0
**Total flux from weak emitters**	**775**	**15.3 ± 5.1**
Erupting volcanoes (2005 to 2017)		
with C/S data available close to eruption	26	1.1
with C/S extrapolated	52	0.7
**Total flux from erupting volcanoes**	**78**	**1.8 ± 0.9**
**Total passive degassing**		**51.3 ± 5.7**
**Total CO**_**2**_**flux from the world’s subaerial volcanoes**		** 53.1 ± 5.8**

The assessment of errors of the eruptive CO_2_ flux requires additional information that is not currently available. For the majority of eruptive SO_2_ measurements, no errors are provided^29^ and the C/S ratios used for computing the CO_2_ emissions are not measured during the eruptions but only prior to the eruptions. Given these uncertainties, we define the error on the eruptive CO_2_ flux as ±50%, emphasizing that it is poorly constrained with present data.

### Preliminary considerations for diffuse CO_2_ emissions

A preliminary estimate of the volcanic-hydrothermal CO_2_ emitted by diffuse emission from soil and lakes is attempted using the published data that are reported in the Magmatic Degassing (MaGa) database at www.magadb.net. The relatively low number of observations with respect to the probable large global number of diffuse degassing structures, hampers the approach of simply summing the catalogued emissions. For this reason, our approach consists of (i) defining the Typical Diffuse CO_2_ Emission (TDCE) from diffuse degassing and (ii) estimating a reasonable number of the volcanoes hosting diffuse degassing structures. We note that many papers treating CO_2_ degassing from volcanoes also include a non-quantified (but possibly  significant) fraction of CO_2_ from background-biogenic sources present. For this reason, we limit the computation of a typical diffuse degassing environment to those articles where the deep-volcanic contribution is clearly differentiated from the biogenic source. Since the published total CO_2_ fluxes catalogued in MaGa include also the contribution from biogenic sources, TDCE is estimated starting from the 73 cases (Table S3) where the ‘volcanic’ CO_2_ output is explicitly separated from the biogenic one. The resulting CO_2_ fluxes, expressed in TgCO_2_/y, show a lognormal distribution with a mean of 1.99 and standard deviation of 0.78. Applying a Monte Carlo approach we estimate that the mean of the data (i.e. our best estimation of TDCE) is 0.18 TgCO_2_/y (0.09–0.33 TgCO_2_/y, 95% confidence interval). Assuming that all the 487 degassing volcanoes host a diffuse degassing structure the total emission from diffuse degassing processes would result in 93 Tg/y (47 Tg/y-174 Tg/y, 95% confidence interval), or 21.22 × 10^11^ mol/y.

## Results and Discussion

Table 2 summarizes the results of the CO_2_ flux calculations. Including both strong  and weak emitters, the total global flux of CO_2_ from passively degassing volcanoes is 51.3 Tg CO_2_/y or 11.7 × 10^11^ mol CO_2_/y. Our value of 15.3 Tg CO_2_/y from weakly emitting volcanoes represents about 30% of the total CO_2_ flux from passively degassing (51.3 Tg CO_2_/y) volcanoes. Notably, our preliminary estimate of diffuse degassing (93 Tg CO_2_/y) is almost twice the total volcanic emission estimate. We stress, however, that this preliminary result could change in the future as the databases of diffuse degassing will include a larger number of data.

### Distribution of CO_2_ fluxes from global emitters and global fluxes of other volatiles

**Figure 4:**
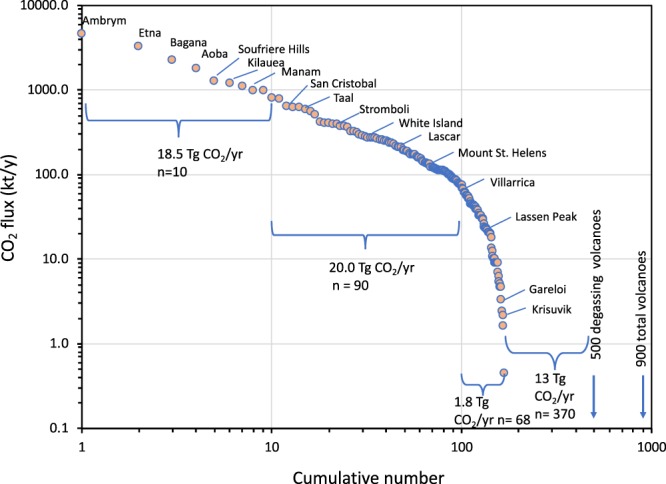
Cumulative number of degassing volcanoes. Values indicate the measured or estimated CO_2_ fluxes of the total 500 degassing volcanoes and the total 900 volcanoes. Data show that the top 100 volcanoes emit about 40 Tg CO_2_/y. The remaining 68 volcanoes for which we have estimates emit only 1.8 Tg CO_2_/y.

Figure 4 shows the rank-order distribution of CO_2_ emitters globally. The compilation of^12^ has approximately 900 volcanoes in total of which about 400 are not degassing, leaving about 500 that have some type of degassing activity. As previously recognized for SO_2_^8,30^ and CO_2_^11,17,31^, a few large emitters contribute the vast majority of emissions to the global volcanic gas flux. Based on our compilation, the top ten volcanoes contribute approximately 18.5 Tg CO_2_/y or 50% of the 40 Tg CO_2_/y emitted by the top 100 volcanoes. This finding is in agreement with the recent estimate of 38.7 ± 2.9 Tg CO_2_/y for the 91 most actively degassing volcanoes presented by^17^. This leaves about 400 volcanoes that have some type of degassing and of these we have emission estimates for 68 volcanoes that emit 1.8 Tg CO_2_/y. The remaining ~370 volcanoes therefore emit about 13 Tg CO_2_/y, considering our total estimate of 51.3 Tg CO_2_ for all passively degassing volcanoes. The results also show that, taken together the 80 small emitters (releasing <100 kt CO_2_/y) for which we have data emit a combined 3.1 Tg CO_2_/y or almost twice the amount of the erupting volcanoes (1.8 Tg CO_2_/y). Therefore, for the most accurate estimates of global emissions, the weak emitters remain at least as significant as the erupting volcanoes. Importantly, weak emitters may become more active and become strong emitters and vice versa, necessitating continued efforts for quantifying volcanic gas emissions from satellite- and ground-based observations.

The new updated volcanic CO_2_ flux compilation also requires an updated estimation of the flux of other volatiles using previously published other compositional ratios (i.e. H_2_O/CO_2_, HCl/CO_2_) of high temperature gases^32^. Future work should consider updating these high temperature gas data, especially for H_2_O but this is beyond the scope of this work. The data compiled on an arc-by-arc basis is shown in Table 3. Note that in Table 3 we include the estimate of the not measured volcanoes. Following the approach described above, we sum up the number of volcanoes for each arc that are inferred to have hydrothermal degassing and assign 3.7 Tg CO_2_/y to these volcanoes (Table 1). Likewise, we sum the volcanoes that are inferred to have magmatic degassing and assign 11.5 Tg CO_2_/y. This amount is then added to the amounts measured for the weak and strong emitter volcanoes for each arc. The data compiled on an arc-by-arc basis is shown in Table 3. For some arcs, we do not have any available gas ratios and cannot calculate H_2_O and HCl fluxes.

**Table 3:** GLobal arc, continental rift and plume passive degassing volatile fluxes based on revised CO_2_ fluxes.

Arc SUM	CO_2_ (Tg/y) strong emitter	CO_2_ (Tg/y) weak emitter	CO_2_ (Tg/y) ascribed mag/hydro	CO_2_ total Tg/y	SO_2_ (Tg/y)	CO_2_ total 10^9^ mol/yr	SO_2_ 10^9^ mol/yr	H_2_O (10^9^ mol/yr)	HCl (10^9^ mol/yr)	H_2_O/CO_2_	HCl/CO_2_
South America	3.16	0.44	2.83	6.44	7.81	146	122	1680	7	11	0.05
CentAm + Mex	3.39	0.52	0.24	4.14	2.05	94	32	3759	14	40	0.15
Alaska + Aleut	0.65	0.64	0.37	1.66	0.72	38	11	1407	17	37	0.44
Kam + Kuriles	1.89	0.28	2.11	4.28	2.18	97	34	5117	25	53	0.26
Japan	1.30	0.22	1.27	2.79	1.53	63	24	18243	72	288	1.14
IBM	0.80	0.00	0.28	1.07	1.07	24	17				
PNG	5.15	0.00	0.25	5.40	3.01	123	47	1958	13	16	0.11
Indonesia	4.11	0.20	3.24	7.55	2.56	172	40	2739	19	16	0.11
Philippines	0.36	0.51	0.24	1.10	0.27	25	4	400	3	16	0.11
Lesser Antilles	1.30	0.00	0.13	1.43	0.47	33	7				
New Zealand	0.38	0.48	0.01	0.87	0.15	20	2				
N and S Vanuatu	7.39	0.00	0.38	7.77	4.50	177	70				
Scotia	0.12	0.00	0.67	0.79	0.15	18	2				
Italy	3.65	0.12	0.00	3.76	0.81	86	13	619	3	7	0.04
**Total Arc**	**34**	**3**	**12**	**49**	**27**	**1115**	**426**	**35923**	**173**		
**Rift & Plume SUM**											
Congo	1.00			1.00	1.29	22.7	20.2	60.56	0.22	2.67	0.01
Tanzania		0.29		0.29		6.6					
Yemen	0.16			0.16	0.04	3.6	0.6				
Ethiopia					0.02					8.04	0.07
Antarctica					0.02						
**Total Rift**	**1.2**	**0.3**		**1.4**	**1.4**	**32.9**	**20.7**	**60.6**	**0.2**		
Plume SUM											
Iceland											
Galapagos		0.14		0.14	0.01	3.3					
Hawaii	1.16			1.16	1.83	26.3	28.6	17.09	0.23	14.75	0.13
Reunion	0.04			0.04	0.09	0.8	1.3				
**Total Plume**	**3.5**	**0.7**		**4.2**	**4.7**	**96.3**	**71.4**	**17.1**	**0.2**		

Notes: Gas ratios are from high temperature arc-by-arc, rift and hot spot fumaroles compilation^32^. Congo gas ratios are from^36^; PNG and Philippine ratios are assumed the same as for Indonesia because no high T data is available.

Hot spot and continental rift passive degassing fluxes are also shown in Table 3. We recognize that there is a potentially large quantity of diffuse CO_2_ degassing from the East African Rift^33,34^ and from caldera-hosted hydrothermal systems^35^, however, the total continental rift volcanic flux is dominated by Nyiragongo and Nyamuragira in the Congo for which gas ratios are also available measured by FTIR in the plume^36^. Likewise, there is significant hot spot CO_2_ flux from low temperature hydrothermal systems^37^, however, the hot spot volcanic flux is dominated by Kilauea. In the future a more rigorous treatment of hot spot and rift CO_2_ degassing, with particular attention to low temperature hydrothermal systems is needed. Overall, our estimates of volcanic degassing from hot spots and rifts are orders of magnitude smaller than volcanic degassing from arcs, entirely consistent with previous assessments^31,38,39^.

### Comparison with other recent volcanic gas flux compilations

Our value of 51.3 ± 5.7 Tg CO_2_/y is at the lower end of the recent evaluation of Werner *et al*.^11^ who estimated 88 ± 21 Tg CO_2_/y for persistently passive degassing volcanoes. Werner *et al*.^11^ calculated and compiled CO_2_ fluxes for a set of 102 volcanoes with direct measurements and 55 OMI-detected volcanoes that emit a total CO_2_ flux of 44 and 27 Tg CO_2_/y, respectively. The difference between our estimate and the Werner *et al*.^11^ estimate is the result of the new treatment of the volcanoes not emitting large quantities of SO_2_. Our new approach considers the type of degassing at each type of volcano and its tectonic setting and based on these parameters, the likely magmatic C/S ratio.

Compared to other recent compilations, our total arc CO_2_ flux of 11 × 10^11^ mol/y is half of Kagoshima *et al*.^38^ who report 22 ± 5 × 10^11^ mol/y based on arc ^3^He fluxes and about 60% of that reported by Fischer^32^ who reports 19 × 10^11^ mol/y based on previously reported arc sulfur fluxes and the same high temperature gas ratios. It is also significantly lower than the global arc CO_2_ flux of Shinohara^40^ who report 120 × 10^11^ mol/y based on detailed studies of the Japan arc and extrapolation to global arcs. However, that study also includes diffuse soil degassing, degassing from springs and emissions during eruptions. If we combine our total flux estimate of 11 × 10^11^ mol/y with our diffuse flux estimate of 21 × 10^11^ mol/y, we obtain 32 × 10^11^ mol/y, still a factor of almost 4 lower than the estimate of Shinohara^40^.

Our maximum estimate of 32 × 10^11^ mol/y for combined diffuse and volcanic CO_2_ degassing includes emission from volcanoes in all tectonic settings. Our low arc fluxes have significant implications for evaluating the global deep carbon cycle. The recent compilation of Kelemen and Manning^41^ suggest significant recycling or storage of C into the deep mantle or below the arc crust, respectively. Our low volcanic CO_2_ emission rates support this idea. Even with the estimated diffuse degassing flux, the total arc CO_2_ flux is on the lower end of most previous estimates suggesting that a significant portion of the incoming C delivered by the subducting plates either gets recycled into the deep mantle^41–43^, is added to the arc crust^41^ and may eventually end up below cratons^44^ or is consumed by microbes and/or trapped in precipitated calcite in the forearc before reaching the zones of magma generation^45^. Compared to the most recent CO_2_ flux estimates from mid ocean ridges (MOR) of 1.32 × 10^12^ mol C/y^46^,our total flux (volcanic craters and diffuse) of 3.2 × 10^12^ mol/y is about a factor of 2.5 higher ,  establishing that subaerial volcanoes are the most significant emitters of volcanic CO_2_ to the Earth's surface.

## Supplementary information

Dataset Table S1

Dataset Table S2

Dataset Table S3

References for supplemental information

## Data Availability

All data are publically available in the EarthChem Library data repository at 10.1594/IEDA/111445. All data are also available in the Tables of the manuscript and in the Supplemental Tables S1–S3.
