# Supplementary material for: The emissions of CO2 and other volatiles from the world’s subaerial volcanoes
Source: Sci Rep. 2019 Dec 10;9:18716. doi: 10.1038/s41598-019-54682-1 (PMC6904619; doi:10.1038/s41598-019-54682-1)
Supplement: Supplementary file 4 — References for supplemental information [file 41598_2019_54682_MOESM4_ESM.docx]

**The emissions of CO_2_ and other volatiles from the world’s subaerial volcanoes**

Tobias P. Fischer^1,*^, Santiago Arellano^2^, Simon Carn^3^, Alessandro Aiuppa^4^, Bo Galle^2^, Patrick Allard^5^, Taryn Lopez^6^, Hiroshi Shinohara^7^, Peter Kelly^8^, Cynthia Werner^8^, Carlo Cardellini^9^, Giovanni Chiodini^10^

^1^ Department of Earth and Planetary Sciences, University of New Mexico, Albuquerque, NM 87131 USA; ^2^ Department of Space, Earth and Environment, Chalmers University of Technology, SE-412 96 Göteborg, Sweden; ^3^ Department of Geological and Mining Engineering Science, Michigan Technological University, Houghton, MI 49931 USA; ^4^ Dipartimento di Scienze della Terra e del Mare (DiSTeM), Università di Palermo, 36 90123 Palermo, Italy ; ^5^ Institut de Physique du Globe de Paris (IPGP), Université de Paris, 75005 Paris, France ; ^6^ Geophysical Institute, Alaska Volcano Observatory, University of Alaska Fairbanks, Fairbanks, AK 99775 USA; ^7^ Geological Survey of Japan, AIST, Tsukuba 305-8567, JAPAN; ^8^ U.S. Geological Survey, David A. Johnston Cascades Volcano Observatory, Vancouver, WA 98683 USA; ^9^ Dipartimento di Fisica e Geologia, Università di Perugia, Perugia, Italy; ^10^ INGV, Sezione di Bologna, Bologna, Italy

*corresponding author : fischer@unm.edu

**References for Table S1**

1 Carn, S. A., Fioletov, V. E., McLinden, C. A., Li, C. & Krotkov, N. A. A decade of global volcanic SO2 emissions measured from space. *Scientific Reports* **7:44095 | DOI: 10.1038/srep44095** (2017).

2 Arellano, S. NOVAC SO2 flux. (in prep).

3 Mori, T. *et al.* Volcanic plume measurements using a UAV for the 2014 Mt. Ontake eruption. . *Earth Planet and Space,* **68**, DOI 10.1186/s40623-40016-40418-40620 (2016).

4 Mori, T. & Kato. Sulfur dioxide emissions during the 2011 eruption of Shinmoedake volcano, Japan. . *Earth, Planet and Space* **65**, 573-580 (2013).

5 Mori, T. *et al.* Time averaged SO2 fluxes of subductionzone volcanoes: Example of a 32-year exhaustive survey for Japanese volcanoes,. *Journal of Geophysical Research: Atmospheres,* **118**, 8662-8674 (2013).

6 Mori, T., Morita, M., Iguchi, M. & Headquarters, F. R. Sulfur dioxide flux monitoring using a regular service ferry after the 2014 eruption of Kuchinoerabujima Volcano, Japan. . *J. Nat. Disaster Sci.,* **38**, 105-118 (2017).

7 Regional Volcanic Observation and Warning Center, S. R. H., JMA Volcanic Activity of Azumayama Volcano (May 2016-September 2016). *Report of Coordination Committee for Prediction of Volcanic Eruption.* **125**, 40-55 (2018).

**References for Table S2**

1 Maldonado, L., Inguaggiato, S., Jaramillo, M., Garzon, G. & Mazot, A. Volatiles and energy released by Puracé volcano. *Bull Volcanol* **79**, DOI: 10.1007/s00445-00017-01168-y (2017).

2 Tamburello, G., Hansteen, T. H., Bredemeyer, S., Aiuppa, A. & Tassi, F. Gas emissions from five volcanoes in northern Chile and implications for the volatiles budget of the Central Volcanic Zone. *Geophys Res. Lett.* **41**, 4961-4969 (2014).

3 Hasselle, N. Gas in volcanic lakes: from dissolved gases to lake gas plumes. . *PhD dissertation, University of Palermo* (2019 ).

4 de Moor, J. M. *et al.* A new sulfur and carbon degassing inventory for the Southern Central American Volcanic Arc: The importance of accurate time-series datasets and possible tectonic processes responsible for temporal variations in arc-scalevolatile emissions. *G-cubed* **DOI 10.1002/2017GC007141** (2017).

5 Battaglia, A. *et al.* Insights into the mechanisms of phreatic eruptions from continuous high frequency volcanic gas monitoring: Rincón de la Vieja volcano, Costa Rica. *Frontiers in Earth Science* **6 (2019)**, 10.3389/feart.2018.00247 (2019).

6 this work

7 Hasselle, N. *et al.* Sulfur Degassing From Steam‐Heated Crater Lakes: El Chichón (Chiapas, Mexico) and Víti (Iceland). *Geophys Res. Lett.* **45**, 7504-7513 (2018).

8 Gudjónsdóttir, S. *et al.* Gas emissions and crustal deformation from the Krýsuvík high temperature geothermal system, Iceland. *J. Volcanol. Geotherm. Res.*, <https://doi.org/10.1016/j.jvolgeores.2018.1004.1007> (2018 (in press)).

**References for Table S3**

1 Chiodini, G. *et al.* Carbon dioxide diffuse emission and thermal energy release from hydrothermal systems at Copahue-Caviahue Volcanic Complex (Argentina). . *Journal of Volcanology and Geothermal Research* **304**, 294-303 doi:doi:10.1016/j.jvolgeores.2015.09.007 (2015). (2015 ).

2 Perez, N. M. e. a. Global CO2 emission from volcanic lakes. . *Geology* **39**, 235-238, doi:doi:10.1130/g31586.1 (2011).

3 Kusakabe, M. e. a. Evolution of CO2 in Lakes Monoun and Nyos, Cameroon, before and during controlled degassing. . *Geochemical Journal* **42**, 93-118, doi:doi:10.2343/geochemj.42.93 (( 2008).

4 Dionis, S. M. e. a. Diffuse CO2 degassing and volcanic activity at Cape Verde islands, West Africa. . *Earth Planets and Space* **67**, doi:doi:10.1186/s40623-015-0219-x (2015).

5 Chiodini, G., Cioni, R., Guidi, M., Raco, B. & Marini, L. Soil CO2 flux measurements in volcanic and geothermal areas. *Appl. Geochem.* **13(5)**, 543–552 (1998).

6 Zhang, L., Guo, Z., Zhang, M. & Cheng, Z. . Study on soil micro-seepage gas flux in the high temperature geothermal area: an example from the Yangbajing geothermal field, South Tibet. . *Acta Petrol. Sin.* **30**, 3612–3626 (2014).

7 Epiard, M. e. a. Relationship between Diffuse CO2 Degassing and Volcanic Activity. Case Study of the Poas, Irazu, and Turrialba Volcanoes, Costa Rica. *Frontiers in Earth Science* **5**, doi:doi:10.3389/feart.2017.00071 (2017).

8 Liegler, A. Diffuse CO2 degassing and the origin of volcabic gas variability from Rincon de la Vieja, Miravalles and Tenorio volcanoes. *Master of Science in Geology thesis, Michigan Technological University,* (2016).

9 Melian, G. e. a. *in American Geophysical Union, Fall Meeting. #V21D-202.* (2010).

10 Padron, E. e. a. Fumarole/plume and diffuse CO2 emission from Sierra Negra caldera, Galapagos archipelago. . *Bulletin of Volcanology* **74**, 1509-1519, doi:doi:10.1007/s00445-012-0610-4 (2012).

11 Padron, E. e. a. Diffuse CO2 emission rate from Pululahua and the lake-filled Cuicocha calderas, Ecuador. . *Journal of Volcanology and Geothermal Research* **176**, 163-169, doi:doi:10.1016/j.jvolgeores.2007.11.023 (2008).

12 Salazar, J. M. L. e. a. Spatial and temporal variations of diffuse CO2 degassing at the Santa Ana-Izalco-Coatepeque volcanic complex, El Salvador, Central America. . . *Special Paper - Geological Society of America* **375**, 135-146 (2004).

13 López, D. L., Ransom, L., Pérez, N. M., Hernández, P. A. & Monterrosa, J. . in Special Paper of the Geological Society of America Vol. 375 (eds W.I. Rose et al.) 191-202 (The Geological Society of America, 2004). *Special Paper of the Geological Society of America* **Vol. 375 (eds W.I. Rose et al.) (The Geological Society of America, 2004).** 191-202 (2004).

14 Hutchison, W., Mather, T. A., Pyle, D. M., Biggs, J. & Yirgu, G. . Structural controls on fluid pathways in an active rift system: A case study of the Aluto volcanic complex. . *Geosphere* **11**, 542-562, doi:doi:10.1130/ges01119.1 (2015).

15 Brombach, T., Hunziker, J. C., Chiodini, G., Cardellini, C. & Marini, L. . Soil diffuse degassing and thermal energy fluxes from the southern Lakki plain, Nisyros (Greece). . *Geophysical Research Letters* **28**, 69-72, doi:doi:10.1029/2000gl008543 (2001).

16 Caliro, S., Chiodini, G., Avino, R., Cardellini, C. & Frondini, F. . Volcanic degassing at Somma-Vesuvio (Italy) inferred by chemical and isotopic signatures of groundwater. . *Applied Geochemistry* **20**, 1060-1076, doi:doi:10.1016/j.apgeochem.2005.02.002 (2005).

17 Cardellini, C., Chiodini, G. & Frondini, F. . Application of stochastic simulation to CO2 flux from soil: Mapping and quantification of gas release. . *Journal of Geophysical Research-Solid Earth* **108**, doi:doi:10.1029/2002jb002165 (2003).

18 Parks, M. M. e. a. Distinguishing contributions to diffuse CO2 emissions in volcanic areas from magmatic degassing and thermal decarbonation using soil gas Rn-222-delta C-13 systematics: Application to Santorini volcano, Greece. . *Earth and Planetary Science Letters* **377**, 180-190, doi:doi:10.1016/j.epsl.2013.06.046 (( 2013).

19 Fridriksson, T. e. a. CO2 emissions and heat flow through soil, fumaroles, and steam heated mud pools at the Reykjanes geothermal area, SW Iceland. . *Applied Geochemistry* **21**, 1551-1569, doi:doi:10.1016/j.apgeochem.2006.04.006 (2006).

20 Hernandez, P. e. a. Diffuse volcanic degassing and thermal energy release from Hengill volcanic system, Iceland. . *Bulletin of Volcanology* **74**, 2435-2448, doi:doi:10.1007/s00445-012-0673-2 (2012).

21 Mazot, A. CO2 degassing and fl uid geochemistry at Papandayan and Kelud volcanoes, Java island, Indonesia *Ph.D. thesis, Université Libre de Bruxelles* (2005).

22 Carapezza, M. L. e. a. Diffuse CO2 soil degassing and CO2 and H2S concentrations in air and related hazards at Vulcano Island (Aeolian arc, Italy). . *Journal of Volcanology and Geothermal Research* **207**, 130-144, doi: doi:10.1016/j.jvolgeores.2011.06.010 (2011).

23 Granieri, D. e. a. Correlated increase in CO2 fumarolic content and diffuse emission from La Fossa crater (Vulcano, Italy): Evidence of volcanic unrest or increasing gas release from a stationary deep magma body? *Geophysical Research Letters* **33**, doi:doi:10.1029/2006gl026460 ( 2006).

24 Inguaggiato, S. e. a. Total CO2 output from Vulcano island (Aeolian Islands, Italy). . *Geochemistry Geophysics Geosystems* **13**, doi:doi:10.1029/2011gc003920 (2012).

25 Inguaggiato, S. e. a. CO2 output discharged from Stromboli Island (Italy). . *Chemical Geology* **339**, 52-60, doi:doi:10.1016/j.chemgeo.2012.10.008 (2013).

26 Camarda, M., De Gregorio, S. & Gurrieri, S. . Magma-ascent processes during 2005-2009 at Mt Etna inferred by soil CO2 emissions in peripheral areas of the volcano. . *Chemical Geology* **330**, 218-227, doi:doi:10.1016/j.chemgeo.2012.08.024 (2012).

27 De Gregorio, S. C., M. . A novel approach to estimate the eruptive potential and probability in open conduit volcanoes. . *Scientific Reports* **6**, doi:doi:10.1038/srep30471 (2016).

28 Giammanco, S., Bellotti, F., Groppelli, G. & Pinton, A. . Statistical analysis reveals spatial and temporal anomalies of soil CO2 efflux on Mount Etna volcano (Italy). . *Journal of Volcanology and Geothermal Research* **194**, 1-14, doi:doi:10.1016/j.jvolgeores.2010.04.006 (2010).

29 Mazot, A. T., Y. CO2 flux from the volcanic lake of El Chichón (Mexico). . *Geofísica Internacional* **48**, 73-83 ( 2009).

30 Favara, R., Giammanco, S., Inguaggiato, S. & Pecoraino, G. . Preliminary estimate of CO2 output from Pantelleria Island volcano (Sicily, Italy): evidence of active mantle degassing. . *Applied Geochemistry* **16**, 883-894, doi:doi:10.1016/s0883-2927(00)00055-x (2001).

31 Caliro, S. e. a. Recent activity of Nisyros volcano (Greece) inferred from structural, geochemical and seismological data. . *Bulletin of Volcanology* **67**, 358-369, doi:doi:10.1007/s00445-004-0381-7 (2005).

32 Frondini, F. e. a. Diffuse CO2 degassing at Vesuvio, Italy. . *Bulletin of Volcanology* **66**, 642-651, doi:doi:10.1007/s00445-004-0346-x (2004).

33 Granieri, D. e. a. Level of carbon dioxide diffuse degassing from the ground of Vesuvio: comparison between extensive surveys and inferences on the gas source. . *Annals of Geophysics* **56**, doi:doi:10.4401/ag-6455 (2013).

34 Chiodini, G. e. a. CO2 degassing and energy release at Solfatara volcano, Campi Flegrei, Italy. . *Journal of Geophysical Research-Solid Earth* **106**, 16213-16221, doi:doi:10.1029/2001jb000246 (2001).

35 Cardellini, C. e. a. Monitoring diffuse volcanic degassing during volcanic unrests: the case of Campi Flegrei (Italy). . *Scientific Reports* **7**, doi: doi:10.1038/s41598-017-06941-2 (2017).

36 Pecoraino, G. e. a. Total CO2 output from Ischia Island volcano (Italy). *Geochemical Journal* **39**, 451-458, doi:doi:10.2343/geochemj.39.451 (2005).

37 Chiodini, G. *et al.* Carbon dioxide earth degassing and seismogenesis in central and southern Italy. *Geophys Res. Lett.* **31**, p. L07615 doi: 07610.01029/02004GL019480., doi:doi: 10.1029/2004GL019480. (2004).

38 Chiodini, G. e. a. Carbon dioxide degassing at Latera caldera (Italy): Evidence of geothermal reservoir and evaluation of its potential energy. . *Journal of Geophysical Research-Solid Earth* **112**, doi:doi:10.1029/2006jb004896 (2007).

39 Hernandez, P. A. e. a. Diffuse emission of CO2 from Miyakejima volcano, Japan. . *Chemical Geology* **177**, 175-185, doi:doi:10.1016/s0009-2541(00)00390-9 (2001).

40 Hernandez, P. A. e. a. Diffuse emission of CO2 from Showa-Shinzan, Hokkaido, Japan: A sign of volcanic dome degassing. . *Pure and Applied Geophysics* **163**, 869-881, doi:doi:10.1007/s00024-006-0038-x (2006).

41 Notsu, K. e. a. Diffuse CO2 efflux from Iwojima volcano, Izu-Ogasawara arc, Japan. . *Journal of Volcanology and Geothermal Research* **139**, 147-161, doi:doi:10.1016/j.jvolgeores.2004.08.003 (2005).

42 Shimoike, Y., Kazahaya, K. & Shinohara, H. . Soil gas emission of volcanic CO2 at Satsuma-Iwojima volcano, Japan. *Earth Planets and Space* **54**, 239-247, doi:doi:10.1186/bf03353023 (2002).

43 Werner, C. C., C. . Comparison of carbon dioxide emissions with fluid upflow, chemistry, and geologic structures at the Rotorua geothermal system, New Zealand. . *Geothermics* **35**, 221-238, doi:doi:10.1016/j.geothermics.2006.02.006 (2006).

44 Salazar, J. M. L. e. a. Diffuse emission of carbon dioxide from Cerro Negro volcano, Nicaragua, Central America. . *Geophysical Research Letters* **28**, 4275-4278, doi:doi:10.1029/2001gl013709 ( 2001).

45 Harvey, M. C., White, P. J., Kenzie, K. M. & Lovelock, B. G. . in New Zealand Geothermal Workshop 2011. **November 2011. 1-8.** (2001).

46 Lewicki, J. L. e. a. Comparative soil CO2 flux measurements and geostatistical estimation methods on Masaya volcano, Nicaragua. . *Bulletin of Volcanology* **68**, 76-90, doi:doi:10.1007/s00445-005-0423-9 (2005).

47 Arpa, M. C. e. a. Geochemical evidence of magma intrusion inferred from diffuse CO2 emissions and fumarole plume chemistry: the 2010-2011 volcanic unrest at Taal Volcano, Philippines. . *Bulletin of Volcanology* **75**, doi:doi:10.1007/s00445-013-0747-9 (2013).

48 Andrade, C., Viveiros, F., Cruz, J. V., Coutinho, R. & Silva, C. Estimation of the CO2 flux from Furnas volcanic Lake (Sao Miguel, Azores). . *Journal of Volcanology and Geothermal Research* **315,** , 51-64, doi:doi:10.1016/j.jvolgeores.2016.02.005 (2016).

49 Pedone, M. e. a. Total (fumarolic plus diffuse soil) CO2 output from Furnas volcano. . *Earth Planets and Space* **67**, doi:doi:10.1186/s40623-015-0345-5 (2015 ).

50 Viveiros, F. e. a. Soil CO2 emissions at Furnas volcano, Sao Miguel Island, Azores archipelago: Volcano monitoring perspectives, geomorphologic studies, and land use planning application. . *Journal of Geophysical Research-Solid Earth* **115**, doi:doi:10.1029/2010jb007555 (2010).

51 Inguaggiato, S., Cardellini, C., Taran, Y. & Kalacheva, E. . The CO2 flux from hydrothermal systems of the Karymsky volcanic Centre, Kamchatka. . *Journal of Volcanology and Geothermal Research* **346**, 1-9, doi:doi:10.1016/j.jvolgeores.2017.07.012 (2017).

52 Hernandez, P. A. e. a. Geochemical evidences of seismo-volcanic unrests at the NW rift zone of Tenerife, Canary Islands, inferred from diffuse CO2 emission. . *Bulletin of Volcanology* **79**, doi:doi:10.1007/s00445-017-1109-9 (2017).

53 Melian, G. e. a. A magmatic source for fumaroles and diffuse degassing from the summit crater of Teide Volcano (Tenerife, Canary Islands): a geochemical evidence for the 2004-2005 seismic-volcanic crisis. . *Bulletin of Volcanology* **74**, 1465-1483, doi: doi:10.1007/s00445-012-0613-1 ((2012).

54 Perez, N. M. e. a. An increasing trend of diffuse CO2 emission from Teide volcano (Tenerife, Canary Islands): geochemical evidence of magma degassing episodes. . *Journal of the Geological Society* **170**, 585-592, doi: doi:10.1144/jgs2012-125 (2013).

55 Melian, G. e. a. Spatial and temporal variations of diffuse CO2 degassing at El Hierro volcanic system: Relation to the 2011-2012 submarine eruption. *Journal of Geophysical Research-Solid Earth* **119**, 6976-6991, doi:doi:10.1002/2014jb011013 (2014).

56 Padron, E. e. a. Dynamics of diffuse carbon dioxide emissions from Cumbre Vieja volcano, La Palma, Canary Islands. . *Bulletin of Volcanology* **77**, doi:doi:10.1007/s00445-015-0914-2 ((2015).

57 Lan, T. F. e. a. Compositions and flux of soil gas in Liu-Huang-Ku hydrothermal area, northern Taiwan. *Journal of Volcanology and Geothermal Research* **165**, 32-45, doi:doi:10.1016/j.jvolgeores.2007.04.015 (2007).

58 Wen, H. Y. e. a. Soil CO2 flux in hydrothermal areas of the Tatun Volcano Group, Northern Taiwan. . *Journal of Volcanology and Geothermal Research* **321**, 114-124, doi:doi:10.1016/j.jvolgeores.2016.04.021 (2016).

59 Bergfeld, D., Goff, F. & Janik, C. J. . Elevated carbon dioxide flux at the Dixie Valley geothermal field, Nevada; relations between surface phenomena and the geothermal reservoir. . *Chemical Geology* **177**, 43-66, doi:doi:10.1016/s0009-2541(00)00381-8 (2001).

60 Bergfeld, D., Evans, W. C., Howle, J. F. & Farrar, C. D. . Carbon dioxide emissions from vegetation-kill zones around the resurgent dome of Long Valley caldera, eastern California, USA. . *Journal of Volcanology and Geothermal Research* **152**, 140-156, doi:doi:10.1016/j.jvolgeores.2005.11.003 (2006).).

61 Bergfeld, D., Evans, W. C., Howle, J. F. & Hunt, A. G. . Magmatic gas emissions at Holocene volcanic features near Mono Lake, California, and their relation to regional magmatism. . *Journal of Volcanology and Geothermal Research* **292**, 70-83, doi:doi:10.1016/j.jvolgeores.2015.01.008 (2015).

62 Evans, W. C., Bergfeld, D., McGimsey, R. G. & Hunt, A. G. . Diffuse gas emissions at the Ukinrek Maars, Alaska: Implications for magmatic degassing and volcanic monitoring. . *Applied Geochemistry* **24**, 527-535, doi:doi:10.1016/j.apgeochem.2008.12.007 (2009).

63 Werner, C. e. a. Decadal-scale variability of diffuse CO2 emissions and seismicity revealed from long-term monitoring (1995-2013) at Mammoth Mountain, California, USA. . *Journal of Volcanology and Geothermal Research* **289**, 51-63, doi: doi:10.1016/j.jvolgeores.2014.10.020 ((2014).
